# Spectrum Sensing for Noncircular Signals Using Augmented Covariance-Matrix-Aware Deep Convolutional Neural Network

**DOI:** 10.3390/s25154791

**Published:** 2025-08-04

**Authors:** Songlin Chen, Zhenqing He, Wenze Song, Guohao Sun

**Affiliations:** 1 Southwest China Institute of Electronic Technology, Chengdu 610036, China; songlinchen605@163.com; 2 School of Aeronautics and Astronautics, Sichuan University, Chengdu 610065, China; sichuandxsongwz@163.com (W.S.); sgh2019@scu.edu.cn (G.S.); 3 Multi-Source Information Intelligent Fusion Key Laboratory of Sichuan Province, Chengdu 610065, China; 4 Robotic Satellite Key Laboratory of Sichuan Province, Chengdu 610015, China

**Keywords:** cognitive radio, convolutional neural network, deep learning, noncircular signal, spectrum sensing

## Abstract

This work investigates spectrum sensing in cognitive radio networks, where multi-antenna secondary users aim to detect the spectral occupancy of noncircular signals transmitted by primary users. Specifically, we propose a deep-learning-based spectrum sensing approach using an augmented covariance-matrix-aware convolutional neural network (CNN). The core innovation of our approach lies in employing an augmented sample covariance matrix, which integrates both a standard covariance matrix and complementary covariance matrix, thereby fully exploiting the statistical properties of noncircular signals. By feeding augmented sample covariance matrices into the designed CNN architecture, the proposed approach effectively learns discriminative patterns from the underlying data structure, without stringent model constraints. Meanwhile, our approach eliminates the need for restrictive model assumptions and significantly enhances the detection performance by fully exploiting noncircular signal characteristics. Various experimental results demonstrate the significant performance improvement and generalization capability of the proposed approach compared to existing benchmark methods.

## 1. Introduction

The increasing demand for high-speed wireless communication has led to a growing need for spectrum resources [1]. However, the traditional static spectrum allocation policy grants exclusive access to licensed spectrum bands for primary users (PUs), leaving secondary users (SUs) unable to utilize these resources, even when they are idle. To address this inefficiency, cognitive radio (CR) technology has been introduced [2]. As a paradigm of software-defined radio, CR offers a promising solution to the spectrum scarcity problem by enabling dynamic spectrum access, thereby accommodating the escalating data rate requirements in wireless communications [3,4]. In fact, CR systems enhance spectral efficiency by opportunistically allowing SUs to access an underutilized spectrum without causing harmful interference to PUs (a concept known as spectrum reuse). To enable this, SUs must continuously perform spectrum sensing, which involves reliably detecting the presence or absence of PUs in a given frequency band. As such, spectrum sensing constitutes a fundamental function of cognitive radio systems and has become a central research topic in both academia and industry [5,6,7].

In the past few years, numerous approaches have been proposed to address the critical challenge of spectrum sensing in CR systems. Among these, energy detection (ED) [8] stands as a mature technique to determine spectrum occupancy by comparing the received signal power against a threshold derived from the prior noise power. However, its performance is highly susceptible to noise uncertainty, due to the dynamic and often unknown nature of noise power in practical environments. Subsequently, maximum eigenvalue detection (MED) [9] emerged by leveraging the eigenvalue properties of a sampled covariance matrix, which also critically relies on prior knowledge of noise variance, making it similarly vulnerable to noise uncertainty [10]. To overcome the reliance on prior noise power estimation and improve performance in non-independent and identically distributed noise environments, several advanced techniques have been developed. Notable examples include eigenvalue moment ratio (EMR) [11], Hadamard ratio detection (HDM) [12], and largest absolute value (LAV) detection [13]. While these methods demonstrate improved robustness against noise uncertainty, they frequently exhibit degraded performance in challenging scenarios such as low signal-to-noise ratio conditions and unstable noise distributions. Importantly, all of the aforementioned approaches are fundamentally model-driven, relying on predefined statistical assumptions and manually designed features. Their effectiveness is thus inherently constrained by the validity of these assumptions.

In contrast to model-driven approaches, deep learning [14,15] has emerged as a powerful paradigm for spectrum sensing, leveraging its capability to autonomously extract discriminative features from raw data, without relying on predefined statistical models. CNNs [16] have been widely adopted due to their proficiency in capturing spatial structures within signal representations. Pioneering this direction, the CM-CNN [17] utilizes a sample covariance matrix (CM) as input to learn environment-adaptive test statistics, and Gao et al. [18] directly processed raw I/Q samples through deep neural networks to exploit their inherent modulation structures. Further innovations have integrated temporal dynamics. For example, the authors in [19] processed both current and historical sensing data, to implicitly model primary user activity patterns, while CNN-LSTM (long short-term memory) [20] combines CNN spatial feature extraction with LSTM sequential modeling to exploit time-dependent signal correlations. For enhanced signal characterization, the method in [21] employs time–frequency representations derived from short-time Fourier transform to capture joint time–frequency features. Recent advances have mainly focused on cross-user interactions and advanced architectures: CNN-Transformer [22] models inter-user signal dependencies through self-attention mechanisms, and channel-attention based parallel CNN-LSTM [23] uses attention-guided spatial feature refinement coupled with parallel temporal modeling.

While the aforementioned methods have demonstrated significant progress, they often implicitly exploit the circularity property of signals. Circular signals exhibit statistical properties that are rotationally invariant in the complex plane. However, not all practical digital modulation schemes produce circular signals. Non-circular (NC) signals, characterized by an asymmetric distribution of their constellation points in the complex plane, are frequently encountered in real-world CR systems. Prominent examples include binary phase shift keying (BPSK), unequal quadrature phase shift keying (UQPSK), and pulse amplitude modulation (PAM), etc. For such signals, valuable discriminatory information resides not only in the conventional covariance matrix, but also in the complementary covariance matrix. In consideration of this, researchers have developed enhanced spectrum sensing techniques specifically designed to leverage non-circularity. For instance, the NC-HDM method [24] extends the conventional HDM test by incorporating a complementary covariance matrix, significantly improving detection for NC signals compared to its circular counterpart. Similarly, the NC-LAV (largest absolute value) algorithm [25] integrates non-circular information into the LAV framework, yielding substantial performance gains over the original LAV method [13] in NC signal environments. Recently, the authors in [26] jointly exploited both covariance matrices for spectrum sensing under uncalibrated antennas. Nevertheless, these NC-enhanced techniques remain model-driven, inheriting fundamental limitations regarding adaptability to diverse and unstable noise environments.

Motivated by the strengths of CNNs for matrix-shaped data structures and the rich statistical structure inherent in non-circular (NC) signals, we propose an augmented covariance-matrix-aware convolutional neural network (ACM-CNN) for spectrum sensing in cognitive radio systems. The key innovation of the proposed method lies in the construction of an augmented covariance matrix that explicitly integrates both standard covariance and complementary covariance matrices. Such a joint representation enables comprehensive exploitation of both the second-order correlation and the non-circular features of the received signal. Unlike previous noncircular based methods that remain constrained by analytical mathematical models, the proposed ACM-CNN method leverages a data-driven approach to automatically learn complex and discriminative patterns embedded in the augmented matrix, thereby improving robustness to unstable noise and low-SNR conditions. Extensive simulation results demonstrate that ACM-CNN significantly outperformed existing state-of-the-art model-driven methods, as well as conventional deep-learning-based techniques, especially in scenarios involving non-circular signals and uncertain noise environments.

The remaining sections of this paper are organized as follows: Section 2 introduces the system model and the problem formulation. Section 3 presents the proposed spectrum sensing method using an augmented covariance-matrix-aware deep CNN. Section 4 evaluates the performance of the proposed algorithm via extensive numerical experiments. Finally, Section 5 concludes the paper.

Notation: We use the superscripts “∗”, “T”, and “H” to denote the conjugate, the transpose, and the conjugate transpose, respectively. For a vector $x\in C^{n}$, we use $x_{i}$ to represent its *i*-th element. For a matrix $X\in C^{m\times n}$, $x_{ij}$ denotes the element at the intersection of its *i*-th row and *j*-th column. We use x∼CN(x,μ;Σ) to denote that x follows real normal and complex circularly symmetric normal distributions with mean μ and covariance Σ, respectively. In addition, we use $j≜\sqrt{-1}$, 0, and E(·) to denote the imaginary unit, the null matrix, and the expectation operation, respectively.

## 2. Signal Model

Let us consider a multi-antenna cognitive radio (CR) scenario, in which a secondary user (SU) with *M* receiving antennas aims to detect the radio spectrum hole of $M_{0}$ primary users (PUs) and seeks to opportunistically use this spectrum when the PUs are idle. Specifically, the SU is attempting to decide if any of the PUs are transmitting based on *N* available observations. Let $x\left(n\right)={\left[x_{1}\left(n\right),x_{2}\left(n\right),\ldots ,x_{M}\left(n\right)\right]}^{T}$ denote the receiving discrete-time sample at time *n* of SU in the CR system. We assume that each PU in the CR system has one antenna, which transmits a signal or does not transmit a signal randomly over a certain spectrum with incumbent licensees. Therefore, there are just two states of the PU (active or inactive). As such, the spectrum sensing problem for a multi-antenna SU can be formulated as a binary hypothesis testing problem:

(1) $$\begin{array}{cc} \; & \;H_{1}:\;x\left(n\right)=Hs\left(n\right)+u\left(n\right),\\ \; & \;H_{0}:\;x\left(n\right)=u\left(n\right), \end{array}$$

where $H_{1}$ stands for the signal-presence hypothesis; $H_{0}$ denotes the signal-absence hypothesis; $H\in C^{M\times M_{0}}$ denotes the channel matrix between the PU and the SU, which is usually unknown but deterministic during the sensing period (coherence time); $s\left(n\right)={\left[s_{1}\left(n\right),s_{2}\left(n\right),...,s_{M_{0}}\left(n\right)\right]}^{T}$ denotes the signal vector emitted by PU at time *n*; $u\left(n\right)={\left[u_{1}\left(n\right),u_{2}\left(n\right),...,u_{M}\left(n\right)\right]}^{T}$ denotes the additive noise vector at time *n*.

We assume that $s_{i}\left(n\right)\;\left(i=1,...,M_{0}\right)$ is exactly a non-circular signal, with its variance being $\sigma_{s_{i}}^{2}=E\left[\left|{s_{i}}^{2}\right|\right]$ and $E\left[{s_{i}}^{2}\right]=\kappa_{i}e^{j\phi_{i}}\sigma_{s_{i}}$ with $\phi_{i}\in \left[-\pi ,\pi \right)$ and $\kappa_{i}\in \left[0,1\right]$ being the noncircularity phase and the noncircularity rate, respectively. Moreover, we assume that the noise ${\left\{u_{i}\left(n\right)\right\}}_{i=1}^{M}$ is statistically independent of each other, independent of the signals, and follows a circular Gaussian distribution, i.e., $E[{u_{i}}^{2}\left(n\right)]=0$ and $u_{i}\left(n\right)∼\mathrm{CN}\left(0,\sigma_{u_{i}}^{2}\right)\left(i=1,\ldots ,M\right)$, where $\sigma_{u_{i}}^{2}=E\left[\left|{u_{i}}^{2}\left(n\right)\right|\right]$ is the noise variance. Note that $\sigma_{u_{i}}^{2}$ is not necessarily equal to $\sigma_{u_{j}}^{2}$ for i≠j in practice, which corresponds to the case of an uncalibrated multiple antenna receiver [26]. As such, the augmented covariance matrix can be expressed as

(2) $$\begin{array}{cc} \; & \underline{R}=E\left[\underline{x}\left(n\right){\underline{x}}^{T}\left(n\right)\right]=\left[\begin{array}{cc} R & \tilde{R}\\ {\tilde{R}}^{*} & R^{*} \end{array}\right] \end{array}$$

where $\underline{x}\left(n\right)={\left[x^{T}\left(n\right),x^{H}\left(n\right)\right]}^{T}$ denotes the augmented signal vector consisting of x(n) and $x^{*}\left(n\right)$ and

(3) $$\begin{array}{c} R=E\left[x\left(n\right)x^{H}\left(n\right)\right],\;\tilde{R}=E\left[x\left(n\right)x^{T}\left(n\right)\right]. \end{array}$$

Herein, R and $\tilde{R}$ stand for the conventional covariance matrix and the complementary covariance matrix, respectively. Note that if the signal vector of PU s(n) is noncircular, then we have $\tilde{R}\neq 0$, otherwise $\tilde{R}=0$. The commonly-used noncircular signals with $\tilde{R}\neq 0$ can be the BPSK, offset QPSK, UQPSK, and minimum shift keying (GMSK) signal, etc.

Now, the spectrum sensing problem at hand is to decide whether the PU signals ${\left\{s_{i}\left(n\right)\right\}}_{i=1}^{M_{0}}$ exist or not from the available *N* noisy observations ${\left\{x\left(n\right)\right\}}_{n=1}^{N}$. To fully exploit the full statistical property of noncircular signals and improve the detection performance, it is crucial to construct a test statistic using the augmented covariance matrix $\underline{R}$. Typical model-driven methods include NC-HDM [24] and NC-LAV [25], which, however, cannot be applied to the case with uncertain or unstable noise. To address this, in the following, we use an augmented covariance-matrix-aware deep CNN, i.e., a data-driven approach, to learn a robust and generalized test statistic for enhancing the spectrum sensing performance of noncircular signals.

## 3. Spectrum Sensing Using Augmented Covariance-Matrix-Aware Deep CNN

**Figure 1:**
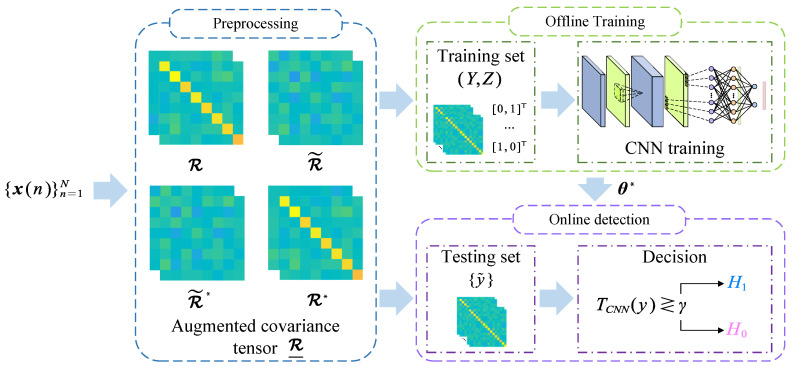
ACM-CNN-based detection framework for spectrum sensing of noncircular signals.

In this section, we propose a deep-learning-based spectrum sensing framework based on augmented covariance-matrix-aware convolutional neural networks, which is illustrated in Figure 1. The proposed framework mainly consists of three stages: data preprocessing, offline training, and online detection, as will be elaborated as follows:

### 3.1. Data Preprocessing

In each sensing period, the SU collects *N* observations ${\left\{x\left(n\right)\right\}}_{n=1}^{N}$ to perform a spectrum sensing. Here, we adopt the full second-order statistical sample covariance matrix of ${\left\{x\left(n\right)\right\}}_{n=1}^{N}$, i.e., the usual sample covariance matrix denoted as $\hat{R}$, while the complementary sample covariance matrix is denoted as $\hat{\tilde{R}}$. Both $\hat{R}$ and $\hat{\tilde{R}}$ are designed as the input of the CNN. The sample covariance matrix $\hat{R}$ and complementary sample covariance matrix $\hat{\tilde{R}}$ from *N* observations are, respectively, calculated as

(4) $$\begin{array}{cc} \; & \hat{R}=\frac{1}{N}\sum_{n=0}^{N-1}x\left(n\right)x^{H}\left(n\right),\\ \; & \hat{\tilde{R}}=\frac{1}{N}\sum_{n=0}^{N-1}x\left(n\right)x^{T}\left(n\right). \end{array}$$

Then, we obtain an augmented sample covariance matrix

(5) $$\begin{array}{cc} \; & \underline{\hat{R}}=\left[\begin{array}{cc} \hat{R} & \hat{\tilde{R}}\\ {\hat{\tilde{R}}}^{*} & {\hat{R}}^{*} \end{array}\right]. \end{array}$$

The reason for using an augmented sample covariance matrix is that it contains not only the energy information but also the correlation information between antennas and between the usual covariance matrix and complementary covariance matrix. To facilitate subsequent processing by CNNs, which typically operate on real-valued inputs, we decompose the complex-valued augmented covariance matrix $\underline{\hat{R}}$ into its real and imaginary components. Specifically, we construct the input of CNNs as

(6) $$\begin{array}{c} \underline{R}=\left[ℜ\left\{\underline{\hat{R}}\right\}\|ℑ\left\{\underline{\hat{R}}\right\}\right]=\left[\begin{array}{cc} R & \tilde{R}\\ {\tilde{R}}^{*} & R^{*} \end{array}\right]\in R^{2M\times 2M\times 2}, \end{array}$$

where ℜ{·} and ℑ{·} denote the real and imaginary components, respectively; and [·‖·] denotes the stacking operation of a matrix to form a three-dimensional tensor. In a word, the two real-valued matrices $ℜ\left\{\underline{\hat{R}}\right\}$ and $ℑ\left\{\underline{\hat{R}}\right\}$ are concatenated along a new dimension to form a three-dimensional augmented covariance tensor $\underline{R}$. Similarly, R, $\tilde{R}$, ${\tilde{R}}^{*}$, and $R^{*}$ represent the tensor formed by stacking the real and imaginary parts of $\hat{R}$, $\hat{\tilde{R}}$, ${\hat{\tilde{R}}}^{*}$, and ${\hat{R}}^{*}$, respectively.

Meanwhile, the binary hypothesis labels are encoded as one-hot vectors to align with the output layer of the CNN classifier:

(7) $$\begin{array}{cc} H_{1} & \to z={\left[1,0\right]}^{T} \end{array}$$

(8) $$\begin{array}{cc} H_{0} & \to z={\left[0,1\right]}^{T}. \end{array}$$

This representation enables the CNN network to output class probabilities directly, where the first element in *z* corresponds to the probability of $H_{1}$ and the second to the probability of $H_{0}$.

**Figure 2:**
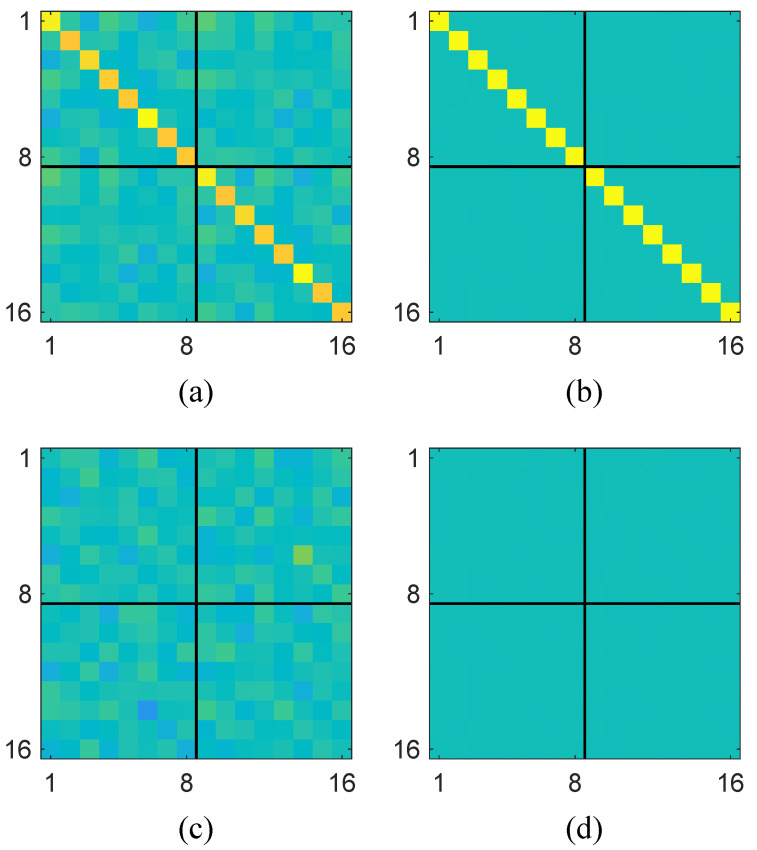
Heatmap comparisons of augmented covariance matrix: (**a**) real part of $\underline{\hat{R}}$ under $H_{1}$, (**b**) real part of $\underline{\hat{R}}$ under $H_{0}$, (**c**) imaginary part of $\underline{\hat{R}}$ under $H_{1}$, (**d**) imaginary part of $\underline{\hat{R}}$ under $H_{0}$.

The unitization of the augmented sample covariance matrix in Equation (5) has significant advantages in spectrum sensing for noncircular PU signals. Specifically, it can contribute to learning more discriminative patterns and a generalized test statistic. To illustrate this, we consider a noncircular signal case with N=100,000 and M=8, where the matrix-shaped heatmaps of the real and imaginary parts of $\underline{\hat{R}}$ under $H_{1}$ and $H_{0}$ are shown in Figure 2 for comparison. Herein, Figure 2a,b represent the real parts of the augmented covariance matrix under the presence and absence of the primary user signal, respectively, while Figure 2c,d show the corresponding imaginary parts. The two block matrices along the main diagonal correspond to the conventional covariance matrices, whereas the off-diagonal blocks represent the complementary covariance matrices (also known as pseudo-covariance). It is seen that the pseudo-covariance matrices (i.e., the off-diagonal blocks) exhibit a clear distinction between the cases with and without the presence of the primary user signal. This indicates that the pseudo-covariance of non-circular signals provides additional discriminative information that can be effectively used to distinguish between signal presence and absence, thereby enabling the learning of a more accurate data-driven test statistic.

### 3.2. Offline Training for Spectrum Sensing of Noncircular Signals

In the offline training stage, the labeled *K* training samples for spectrum sensing are collected to construct the training set:

(9) $$\begin{array}{c} \left(Y,Z\right)=\{\left(y^{\left(1\right)},z^{\left(1\right)}\right),\left(y^{\left(2\right)},z^{\left(2\right)}\right),\ldots ,\left(y^{\left(K\right)},z^{\left(K\right)}\right)\} \end{array}$$

where $\left(y^{\left(k\right)},z^{\left(k\right)}\right)$ denotes the k-th(k=1,2,…,K) training sample of the training set (Y,Z). For a single example (y,z), *y* represents the input data for the neural network, which is the augmented covariance matrix calculated by Equation (5). We note that $z\in \{{\left[0,1\right]}^{T},{\left[1,0\right]}^{T}\}$ represents the corresponding label of $H_{0}$ and $H_{1}$. The output of CNN is formulated as a normalized class score vector:

(10) $$\begin{array}{c} h_{\theta }\left(y^{\left(k\right)}\right)=\left[\begin{array}{c} h_{\theta |H_{1}}\left(y^{\left(k\right)}\right)\\ h_{\theta |H_{0}}\left(y^{\left(k\right)}\right) \end{array}\right], \end{array}$$

where $h_{\theta |H_{1}}\left(y^{\left(k\right)}\right)+h_{\theta |H_{0}}\left(y^{\left(k\right)}\right)=1$. We note that $h_{\theta }\left(\cdot \right)$ is a mapping from $y^{\left(k\right)}$ to $z^{\left(k\right)}$ with CNN parameters θ. In this case, $h_{\theta |H_{i}}\left(y^{\left(k\right)}\right)$ represents the class score under $H_{i}$. Because the output of CNN behaves like a probability distribution, we can define the loss function L(θ) using cross-entropy as

(11) $$\begin{array}{cc} L(\theta ) & ≜-\frac{1}{K}\sum_{k=1}^{K}\left(z_{1}^{\left(k\right)}\log h_{\theta |H_{1}}\left(y^{\left(k\right)}\right)+\left(1-z_{1}^{\left(k\right)}\right)\log(1-h_{\theta |H_{1}}\left(y^{\left(k\right)}\right))\right) \end{array}$$

where $z_{1}^{\left(k\right)}$ denotes the first element of $z^{\left(k\right)}$. A smaller loss function value L(θ) indicates that the output of CNN, i.e., $h_{\theta }\left(y^{\left(k\right)}\right)$, and the label $z^{\left(k\right)}$ tend to be closer. Our objective is to derive the optimal parameter $\theta^{*}$ that minimizes the loss function L(θ), that is

(12) $$\begin{array}{c} \theta^{*}=\underset{\theta }{\arg\min}L(\theta ). \end{array}$$

However, obtaining a closed-form analytical solution for L(θ) is intractable. We therefore employ gradient descent methods to compute a suboptimal solution. Based on Bayesian criterion, we have

(13) $$\begin{array}{cc} \; & P\left(y|H_{1}\right)=\frac{P\left(H_{1}|y\right)\cdot P\left(y\right)}{P(H_{1})}=\frac{h_{\theta^{*}|H_{1}}\left(y\right)\cdot P\left(y\right)}{P(H_{1})}\\ \; & P\left(y|H_{0}\right)=\frac{P\left(H_{0}|y\right)\cdot P\left(y\right)}{P(H_{0})}=\frac{h_{\theta^{*}|H_{0}}\left(y\right)\cdot P\left(y\right)}{P(H_{0})}, \end{array}$$

where $P(y|H_{i})$ denotes the conditional probability given $H_{i}$, P(y) is the marginal probability, and $P(H_{i})$ indicates the priori probability of $H_{i}$ in the training process.

According to the Neyman–Pearson theorem, we can obtain a CNN-based likelihood ratio test:

(14) $$\begin{array}{c} T_{\mathrm{CNN}}\left(y\right)=\frac{h_{\theta^{*}|H_{1}}\left(y\right)}{h_{\theta^{*}|H_{0}}\left(y\right)}\cdot \frac{P(H_{0})}{P(H_{1})}=\frac{h_{\theta^{*}|H_{1}}\left(y\right)}{h_{\theta^{*}|H_{0}}\left(y\right)}≷\gamma , \end{array}$$

where the test statistic $T_{\mathrm{CNN}}\left(y\right)$ denotes the CNN-based likelihood ratio, and the threshold γ can be derived from the false alarm constraint. For the convenience of analysis, we set $P\left(H_{1}\right)=P\left(H_{0}\right)=0.5$ in the training process.

### 3.3. Covariance-Matrix-Aware CNN Structure

Note that CNNs exhibit strong capabilities for extracting discriminative features from tensor-shaped data, such as images. Motivated by this property, we leverage CNNs to extract informative features from a tensor-shaped representation $\underline{R}$ for spectrum sensing of noncircular signals.

**Figure 3:**
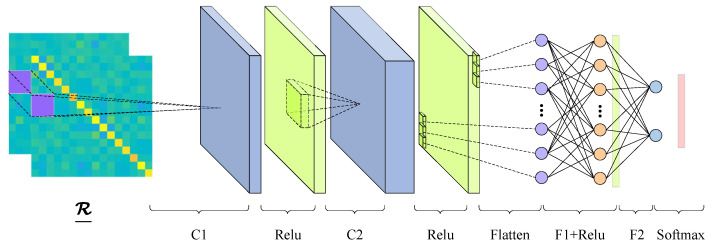
Proposed CNN structure for spectrum sensing of noncircular signals.

We design a CNN-based architecture composed of two convolutional layers, followed by two fully connected layers, as illustrated in Figure 3. Specifically, $C_{i}\;\left(1\leq i\leq 2\right)$ and $F_{i}\;\left(1\leq i\leq 2\right)$ denote the *i*-th convolutional and fully connected layers, respectively. Each convolutional layer is followed by a rectified linear unit (ReLU) activation function to introduce non-linearity and enhance the representational capacity of the network. The first convolutional layer $C_{1}$ is responsible for capturing local spatial features and correlations in $\underline{R}$, while the second layer $C_{2}$ further integrates and abstracts higher-level semantic features from the previous representations. The output of the convolutional layers is then reshaped into a one-dimensional feature vector via a flattening operation, which is subsequently passed to the first fully connected layer $F_{1}$, with ReLU activation for further high-level reasoning. The final fully connected layer $F_{2}$ generates a two-dimensional output vector, which is passed through a softmax function to obtain the posterior probability of each class.

It is worth noting that, unlike typical CNN designs, we do not adopt pooling layers in the proposed CNN architecture. Although pooling operations such as max-pooling or average-pooling can reduce the spatial resolution and computational cost, they inevitably lead to the loss of fine-grained feature details. In spectrum sensing, the presence or absence of weak signals may rely on subtle variations in local structures, and retaining full spatial information is essential to ensure reliable detection performance. Therefore, our structure maintains the original resolution in the convolutional feature maps to preserve as much discriminative information as possible.

### 3.4. Online Detection for Spectrum Sensing of Noncircular Signals

For online detection, the multi-antenna SU collects an unlabeled sample $\tilde{y}$ and then sends $\tilde{y}$ to the well-trained CNN model $h_{\theta^{*}}\left(\cdot \right)$ of (14) to obtain the following test statistic:

(15) $$\begin{array}{c} T_{\mathrm{CNN}}\left(\tilde{y}\right)=\frac{h_{\theta^{*}|H_{1}}\left(\tilde{y}\right)}{h_{\theta^{*}|H_{0}}\left(\tilde{y}\right)}\overset{H_{1}}{\underset{H_{0}}{≷}}\gamma . \end{array}$$

Once we have obtained the test statistic $T_{\mathrm{CNN}}\left(\tilde{y}\right)$, we can quickly make a decision by comparing it with a preset threshold γ. The selection of the threshold γ is determined using the desired probability of false alarm $P_{f}$. The procedure for how to obtain the threshold γ is detailed in Algorithm 1. In addition, for clarification, we summarize the above overall spectrum sensing procedure for noncircular signals in Algorithm 2, which is referred to as the augmented covariance matrix CNN (ACM-CNN) algorithm, where $I_{\max}$ is the maximum number of iterations.
**Algorithm 1** Selection of threshold γ using desired probability of false alarm $P_{f}$1:Construct $Y_{0}=\left\{y^{\left(k\right)}|z^{\left(k\right)}=0,\;1\leq k\leq K\right\}$ from the training dataset $\left(Y,Z\right)=\{\left(y^{\left(1\right)},z^{\left(1\right)}\right),\left(y^{\left(2\right)},z^{\left(2\right)}\right),\ldots ,\left(y^{\left(K\right)},z^{\left(K\right)}\right)\}$, where $z^{\left(k\right)}=0$ indicates that the *k*-th training sample is generated under $H_{0}$, and $K_{0}$ denotes the number of samples of $Y_{0}$ that satisfy $z^{\left(k\right)}=0,\;\forall \;k\in \left\{1,\ldots ,K\right\}$, in *Y*;2:**for**$\;k_{0}=1$ to $K_{0}$ **do**3: Compute the test statistic $T_{\mathrm{CNN}}\left(y_{0}^{\left(k_{0}\right)}\right)=\frac{h_{\theta^{*}|H_{1}}\left(y_{0}^{\left(k_{0}\right)}\right)}{h_{\theta^{*}|H_{0}}\left(y_{0}^{\left(k_{0}\right)}\right)}$ where $y_{0}^{\left(k_{0}\right)}\in Y_{0}$;4:**end for**5:Sort the $K_{0}$ test statistics $\left\{T_{\mathrm{CNN}}\left(y_{0}^{\left(k_{0}\right)}\right)\right\}$ in ascending order as $T_{\mathrm{CNN}}^{\left(1\right)}\leq T_{\mathrm{CNN}}^{\left(2\right)}\leq \ldots \leq T_{\mathrm{CNN}}^{\left(K_{0}\right)}$;6:Determine the index corresponding to $P_{f}$ as per $k^{*}=\left\lfloor \left(1-P_{f}\right)K_{0}\right\rfloor$ where $\left\lfloor \cdot \right\rfloor$ denotes rounding to the nearest integer;7:Set the decision threshold: $\gamma =T_{\mathrm{CNN}}^{\left(k^{*}\right)}$.

**Algorithm 2** ACM-CNN

**% Data Preprocessing**

1:Acquire discrete-time observation signals ${\left\{x\left(n\right)\right\}}_{n=1}^{N}$ by sampling the received signals from *M* antennas;2:Compute the augmented covariance tensor $\underline{R}$ based on ${\left\{x\left(n\right)\right\}}_{n=1}^{N}$ using Equations (4)–(6);

**% Offline Training Phase**

1:Construct the labeled training dataset $\left(Y,Z\right)=\{\left(y^{\left(1\right)},z^{\left(1\right)}\right),\left(y^{\left(2\right)},z^{\left(2\right)}\right),\ldots ,\left(y^{\left(K\right)},z^{\left(K\right)}\right)\}$;2:Initialize the CNN model parameters $\theta^{\left(0\right)}$ and set iteration counter i=0;3:**for** i=1 to $I_{\max}$ **do**4:   Compute the loss function $L(\theta^{\left(i\right)})$ using Equation (11);5:   Update the model parameters $\theta^{\left(i\right)}$ using the Adam optimizer [27];6:
**end for**
7:Obtain the final model parameters $\theta^{*}\leftarrow \theta^{\left(i\right)}$;

**% Online Detection Phase**

1:Set the decision threshold γ>0 according to Algorithm 1;2:Acquire unlabeled test data $\tilde{y}$;3:Input $\tilde{y}$ into the trained CNN model to obtain the predicted result $\tilde{z}$;4:Make spectrum occupancy decision by comparing each $\tilde{z}$ with threshold γ: decide $H_{1}$ (occupied) if $\tilde{z}>\gamma$, otherwise decide $H_{0}$ (idle), as described in Equation (15).


## 4. Simulation Results

This section presents numerical experiments to corroborate the performance of the proposed ACM-CNN algorithm. We consider a multi-antenna CR system, where a PU randomly chooses to transmit a non-circular signal or not using $M_{0}=4$ antennas, and a SU equipped with M=8 antennas receives the PU signals and attempts to determine whether the PU is active or inactive. The transmitted signals are noncircularity and complex Gaussian distributed, i.e., $s_{k}\left(n\right)∼\mathrm{CN}\left(0,\sigma_{s}^{2}\right)$ ($k=1,\ldots ,M_{0}$), and the noncircularity rate is given as $\kappa =\left|\frac{E[s_{k}{\left(n\right)}^{2}]}{E[|s_{k}\left(n\right){|}^{2}]}\right|$. Except for the last experiment to explore the detection performance of different methods versus noncircularity rate κ, the rest of the numerical experiments are carried out under the condition of κ=0.99. The noises for the different receive antennas ${\left\{u_{m}\left(n\right)\right\}}_{m=1}^{M}$ are statistically independent and also independent of the PU signals. Specifically, the noises are generalized according to

(16) $$\left[\begin{array}{c} u_{1}\left(n\right)\\ u_{2}\left(n\right)\\ \ldots \\ u_{M}\left(n\right) \end{array}\right]=\left[\begin{array}{cccc} \sigma_{a_{1}} & \; & & \\ \; & \sigma_{a_{2}} & \; & \\ \; & \; & \ddots & \\ \; & \; & & \sigma_{a_{K}} \end{array}\right]\left[\begin{array}{c} u_{1}^{0}\left(n\right)\\ u_{2}^{0}\left(n\right)\\ \ldots \\ u_{M}^{0}\left(n\right) \end{array}\right]\left[\begin{array}{cccc} \sigma_{t_{1}} & \; & & \\ \; & \sigma_{t_{2}} & \; & \\ \; & \; & \ddots & \\ \; & \; & & \sigma_{t_{N}} \end{array}\right],$$

where $u_{m}^{0}\left(n\right)$ is a standard circularly symmetric complex Gaussian noise, i.e., $u_{m}^{0}\left(n\right)∼\mathrm{CN}\left(0,1\right)$, and $\sigma_{a_{m}}^{2}$ and $\sigma_{t_{n}}^{2}$ indicate the noise variances across antennas and time, respectively. Furthermore, the logarithmic values of $\sigma_{a_{m}}^{2}$ and $\sigma_{t_{n}}^{2}$ are assumed to follow uniform distributions to construct the noise uncertainty. Specifically, they are defined as

(17) $$\begin{array}{c} 10\log_{10}\sigma_{a_{m}}^{2}∼U\left(-\varepsilon_{a},\varepsilon_{a}\right),\\ 10\log_{10}\sigma_{t_{n}}^{2}∼U\left(-\varepsilon_{t},\varepsilon_{t}\right). \end{array}$$

where U(a,b) denote the uniform distributions from *a* to *b*. Meanwhile, $\varepsilon_{a}$ and $\varepsilon_{a}$ represent the uncertainty factors of the noise variance for the antennas and time, respectively, which are used to characterize the degree of fluctuation of the noise power in decibels (dB). The signal-to-noise ratio (SNR) is defined as

(18) $$\begin{array}{c} \mathrm{SNR}≜10\log_{10}\frac{MN\sigma_{s}^{2}}{\sum_{m=1}^{M}\sigma_{a_{m}}^{2}\sum_{n=1}^{N}\sigma_{t_{n}}^{2}} \end{array}$$

where *N* is the number of samples. The channel matrix H between the PU and SU is modeled as a Rician fading channel, that is

(19) $$\begin{array}{c} H≜\sqrt{\frac{\alpha }{\alpha +1}}\;H_{\mathrm{LOS}}+\sqrt{\frac{1}{\alpha +1}}\;H_{\mathrm{NLOS}}, \end{array}$$

where α denotes the Rician factor, representing the power ratio between the deterministic line-of-sight (LOS) component $H_{\mathrm{LOS}}$ and the scattered non-line-of-sight (NLOS) component $H_{\mathrm{NLOS}}$. Each element in $H_{\mathrm{LOS}}$ or $H_{\mathrm{NLOS}}$ is assumed to follow a standard complex Gaussian distribution, i.e., CN(0,1). The LOS component is considered quasi-static, while the NLOS component varies randomly over time to reflect realistic multipath fading. In the simulations, α is set to 100.

For comparison, we selected several typical spectrum sensing algorithms as benchmarks, which included the covariance-matrix-aware convolutional neural network (CM-CNN) method [17], noncircular local average variance (NC-LAV) [25], local average variance (LAV) [13], noncircular-based Hadamard (NC-HDM) ratio test [24], Hadamard (HDM) ratio test [12], eigenvalue moment ratio (EMR) [11], and energy detection (ED) [8]. All simulation results were obtained by averaging 60,000 Monte Carlo realizations. There were additional 35,000 training samples for the data-driven methods, including the proposed ACM-CNN and the CM-CNN [17]. The hyperparameters of the augmented covariance matrix based CNN in our simulations are given in Table 1. The simulations were conducted on a personal computer equipped with an Intel Core i7-13800H CPU (Intel Corporation, Santa Clara, CA, USA) and an NVIDIA RTX 2000 Ada Generation Laptop GPU (NVIDIA Corporation, Santa Clara, CA, USA). It should be noted that the online detection time per sample was approximately 0.0398 seconds by averaging 1000 trials, thereby supporting applicability to real-time detection in practical scenarios.

### 4.1. ROC Detection Performance Comparison

First, we evaluate the detection performance of the proposed method by plotting receiver operating characteristic (ROC) curves, which illustrate the probability of detection versus the probability of false alarm. Three sets of experiments were conducted: (i) i.i.d. noise; (ii) varying the noise variance in antennas ($\varepsilon_{a}\neq 0$) while the keeping noise variance fixed in time ($\varepsilon_{t}=0$); and (iii) varying the noise variance in time ($\varepsilon_{t}\neq 0$) while keeping the noise variance in antennas fixed ($\varepsilon_{a}=0$). For each case, we evaluated the detection performance under different numbers of observed samples *N*. In this case, the SNR was fixed at −18dB.

**Figure 4:**
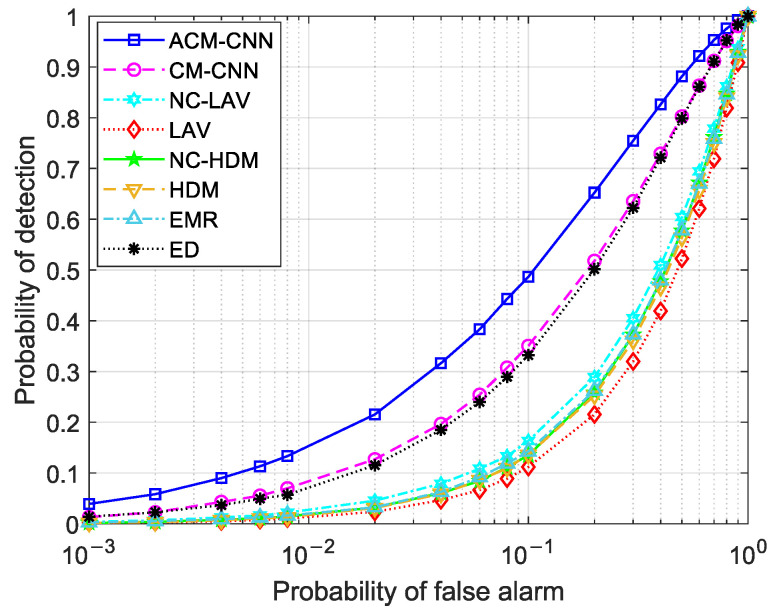
ROC curves of different algorithms: N=40, SNR =−18 dB, $\varepsilon_{a}=0$ dB, $\varepsilon_{t}=0$ dB.

**Figure 5:**
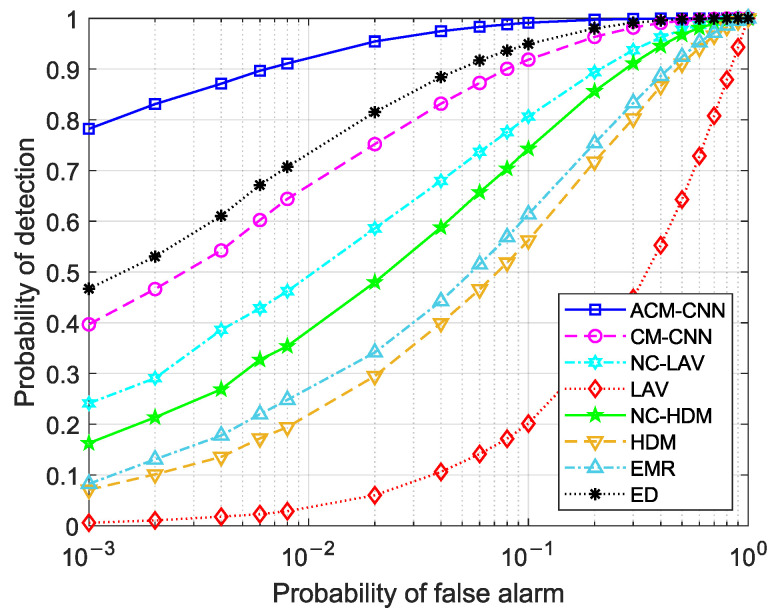
ROC curves of different algorithms: N=360, SNR =−18 dB, $\varepsilon_{a}=0$ dB, $\varepsilon_{t}=0$ dB.

**Figure 6:**
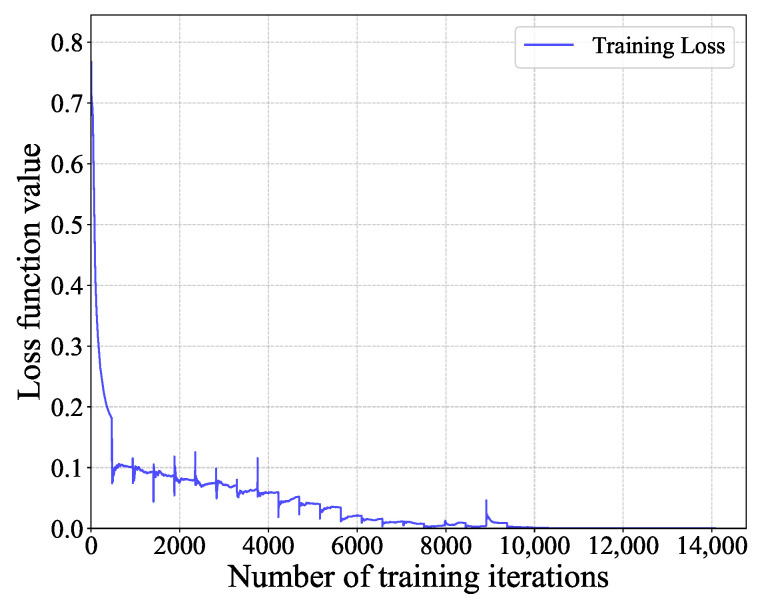
Training loss function value versus the number of training iterations.

Figure 4 and Figure 5 show the ROC curves of the different methods under i.i.d. noise with a low sample size (N=40) and a high sample size (N=360), respectively. It can be observed that our method consistently outperformed all other methods, regardless of the sample size. Specifically, in Figure 4, our ACM-CNN method achieved a detection probability close to 50% under the probability of false alarm $P_{f}=0.1$, which was more than 15 percentage points higher than both CM-CNN and ED. The remaining methods almost failed under such a low-sample environment. In Figure 5, all methods were able to detect under the high-sample environment; however, our method still achieved the best detection performance. It is worth noting that the ED method benefits from prior knowledge of the averaging noise power. Except for ED, all other methods operate without any prior information, i.e., totally blind detection methods. Among these totally-blind detection methods, it is seen that the data-driven approaches outperformed the model-driven ones. Furthermore, we see that the methods that exploit noncircularity demonstrated superior performance when compared to their counterparts that do not use the noncircularity (e.g., NC-HDM outperformed HDM). In order to verify the effectiveness and convergence of the proposed model during training, we also plot the training loss versus the number of training iterations of the ACM-CNN in Figure 6, where the parameter settings are kept the same as those of Figure 5. It is seen that the loss value gradually decreased as the number of training iterations was increased, and eventually converged to a value close to zero. This demonstrates that the trained model was able to effectively learn discriminative features from the data and converged to a stable training state.

**Figure 7:**
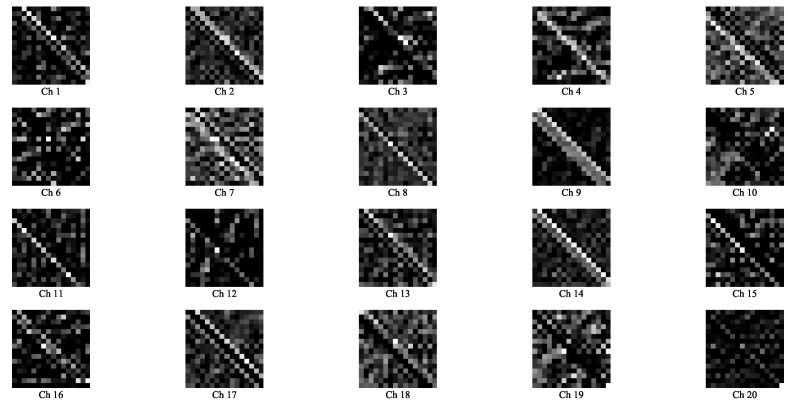
Visualization of the feature maps in C1 layers.

**Figure 8:**
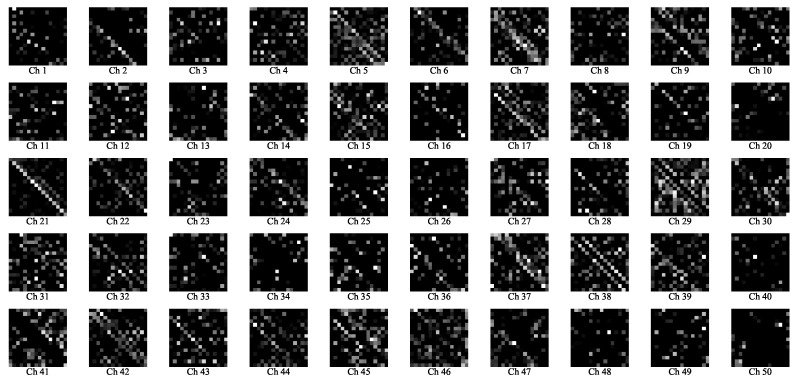
Visualization of the feature maps in C2 layers.

To further understand how the proposed ACM-CNN model extracts discriminative features for spectrum sensing, we visualized the intermediate feature maps of the convolutional layers under hypothesis $H_{1}$. Specifically, the augmented covariance tensor $\underline{R}$ with sample size N=160 was input into the trained model, and a forward pass was performed. The output feature maps of C1 and C2 are normalized and visualized in Figure 7 and Figure 8, respectively. In the figures, “Ch *q*” denotes the *q*-th channel of the output feature map.

From the visualizations, it can be observed that the network effectively captured both the circular and noncircular components embedded in $\underline{R}$. Specifically, the two block matrices along the main diagonal encode the conventional covariance structure, which reflects circular signal characteristics (e.g., Ch 9 in Figure 7 and Ch 21 in Figure 8), while the off-diagonal blocks represent the complementary covariance, which arises exclusively in noncircular signals (e.g., Ch 10 in Figure 7 and Ch 20 in Figure 8). These results demonstrate that the proposed ACM-CNN is capable of jointly exploiting both circular and noncircular statistical features, thereby enabling the learning of more discriminative and robust data-driven test statistics for spectrum sensing.

**Figure 9:**
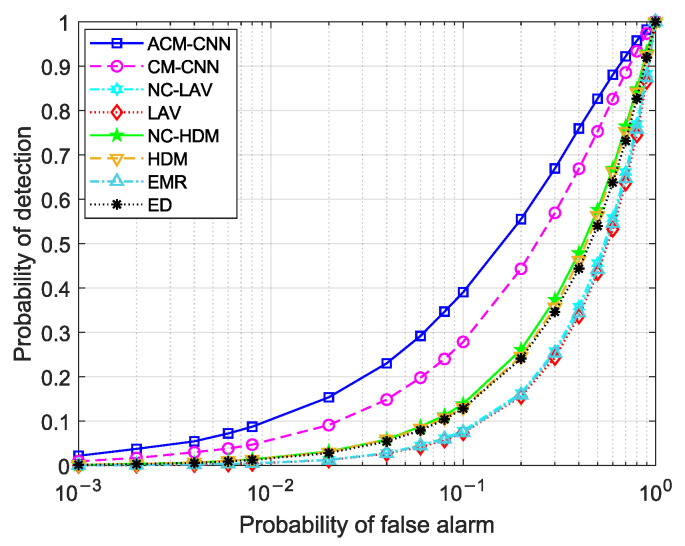
ROC curves of different algorithms: N=40, SNR =−18 dB, $\varepsilon_{a}=5$ dB, and $\varepsilon_{t}=0$ dB.

**Figure 10:**
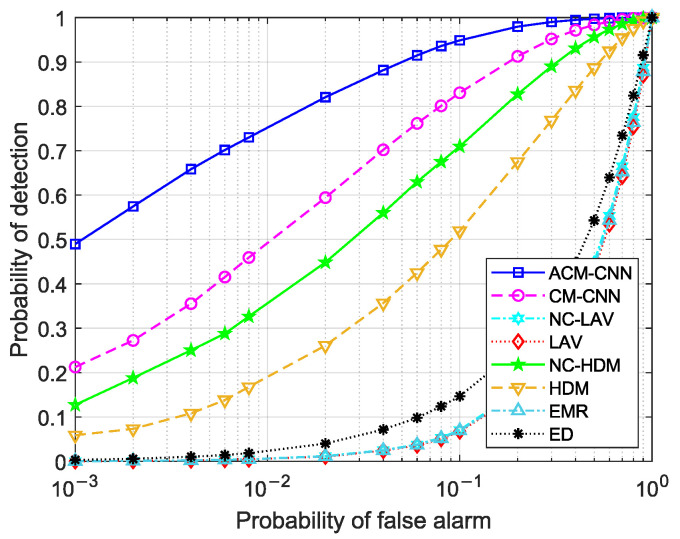
ROC curves of different algorithms: N=360, SNR =−18 dB, $\varepsilon_{a}=5$ dB, and $\varepsilon_{t}=0$ dB.

Figure 9 and Figure 10 illustrate the ROC curves under the condition of $\varepsilon_{a}\neq 0$ for N=40 and N=360, respectively. It can be observed that several methods failed in the presence of unstable noise across the antennas (e.g., ED and EMR), while only the data-driven methods (ACM-CNN and CM-CNN) and those based on the Hadamard ratio test (HDM and NC-HDM) remained functional in such scenarios. However, Hadamard-based methods only achieved satisfactory performance under large sample size conditions. In contrast, the data-driven methods not only performed better overall, but also maintained effectiveness even in the low-sample environment.

By comparing Figure 4 and Figure 9, as well as Figure 5 and Figure 10, we can see that ACM-CNN, CM-CNN, HDM, and NC-HDM exhibited some performance degradation under unstable noise across antennas. Nevertheless, the decline was within an acceptable range, indicating that these four methods exhibited a certain degree of robustness to unstable noise. Our method still achieved the best detection performance under all tested conditions.

**Figure 11:**
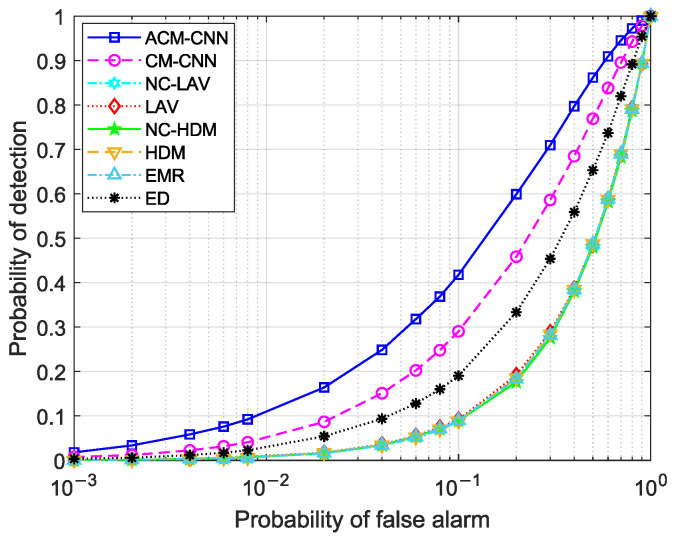
ROC curves of different algorithms: N=40, SNR =−18 dB, $\varepsilon_{a}=0$ dB, $\varepsilon_{t}=5$ dB.

**Figure 12:**
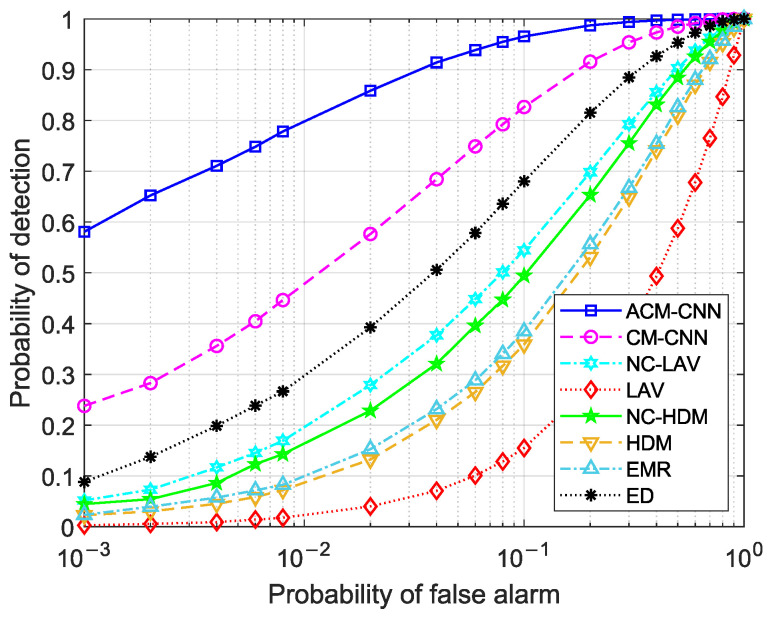
ROC curves of different algorithms: N=360, SNR =−18 dB, $\varepsilon_{a}=0$ dB, $\varepsilon_{t}=5$ dB.

Figure 11 and Figure 12 illustrate the ROC curves under the condition of $\varepsilon_{t}\neq 0$ for N=40 and N=360, respectively. By comparing Figure 4 and Figure 11, as well as Figure 5 and Figure 12, we can observe that all methods experienced some performance degradation under temporally unstable noise, indicating that they all exhibited a certain degree of robustness to such noise. Compared to the unstable noise across antennas, the adverse impact of temporally unstable noise was relatively smaller. In Figure 12, ACM-CNN achieved a detection probability exceeding 96% at a false alarm rate $P_{f}=0.1$, significantly outperforming all other methods.

### 4.2. Probability of Detection Versus the Number of Observations and SNR

**Figure 13:**
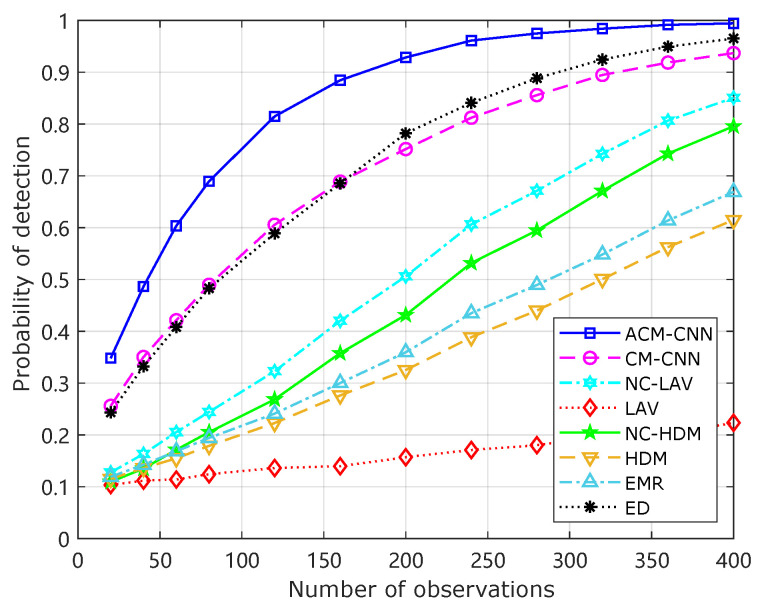
Detection probability versus the number of samples under $P_{f}=0.1$, SNR =−18 dB, $\varepsilon_{a}=0$ dB, $\varepsilon_{t}=0$ dB.

Figure 13 shows how the detection probability of the respective algorithms changed with the number of samples *N* when the probability of false alarm was fixed at 0.1. It can be seen that under an i.i.d. noise environment, the detection performance of all algorithms improved as the number of samples *N* increased. Our ACM-CNN algorithm was the best among all algorithms for all sample numbers *N*.

**Figure 14:**
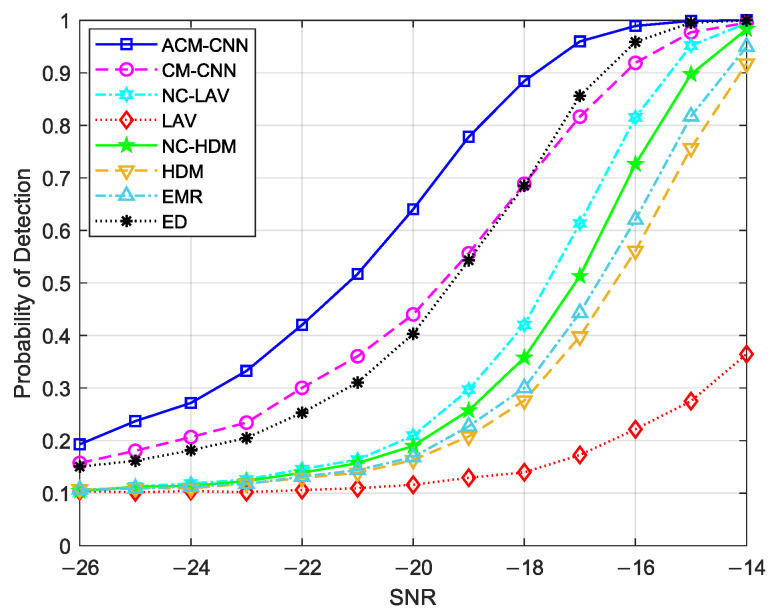
Detection probability versus SNR under $P_{f}=0.1$, N =160, $\varepsilon_{a}=0$ dB, $\varepsilon_{t}=0$ dB.

Figure 14 shows a curve of the detection probability changing with SNR under an i.i.d. noise environment with fixed $P_{f}=0.1$. It can be seen that, under all SNR conditions, our proposed ACM-CNN algorithm was optimal.

Figure 13 illustrates the probability of detection versus number of samples *N* for the various detection algorithms under an i.i.d. noise environment, with the probability of false alarm fixed at 0.1. The results demonstrate that as *N* increased, all methods achieved a progressively better detection performance, which is expected, since more samples provide richer statistical information for signal detection. Among all the evaluated algorithms, our proposed ACM-CNN significantly outperformed the other methods across the entire range of sample numbers. In particular, ACM-CNN exhibited a steeper performance gain with increasing *N*, reaching a detection probability of more than 90% at a number of samples about 175. Model-driven approaches like LAV and HDM performed substantially worse, showing limited improvement with an increasing number of samples, which highlights their insufficiency for leveraging high-dimensional data patterns compared to deep-learning-based methods.

Figure 14 shows the probability of detection versus SNR under an i.i.d. noise assumption and fixed false alarm rate ($P_{f}=0.1$). The results clearly indicate that ACM-CNN consistently achieved the highest detection probability at every SNR level. Especially in the low-SNR regime (e.g., SNR < −20 dB), ACM-CNN exhibited a remarkable performance, whereas the other methods failed to maintain effective performance. As the SNR improved, all algorithms began to benefit, but the performance gap between ACM-CNN and the rest remained evident, underscoring its superior capacity for feature extraction and classification.

### 4.3. Generalizability

Since the proposed ACM-CNN method is data-driven, the deployed neural network model was trained offline based on the data distribution in the training set. However, during online detection, the noise model, SNR, and other environmental factors may differ from those present in the training phase. Therefore, it is meaningful to investigate the generalizability of the proposed method under conditions mismatched between training and testing. Specifically, we evaluate the generalization performance of ACM-CNN by presenting its ROC curves under three scenarios: mismatched SNRs, different degrees of antenna noise uncertainty, and different degrees of time noise uncertainty between training and testing, respectively.

**Figure 15:**
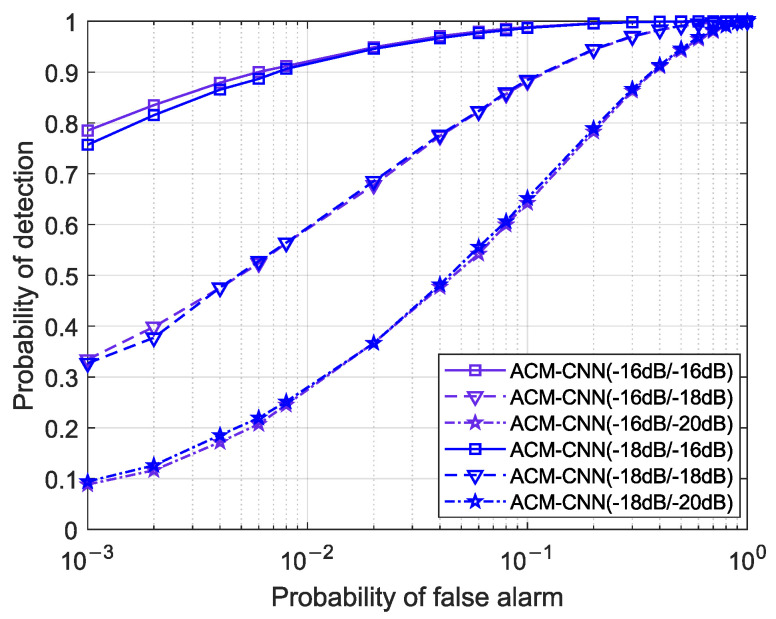
ROC curves with mismatched SNRs between training and detection under N=160, $\varepsilon_{a}=0$ dB, $\varepsilon_{t}=0$ dB.

Figure 15 illustrates the ROC curves of the proposed ACM-CNN method under SNR conditions mismatched between training and detection. Specifically, “ACM-CNN (pdB/qdB)” denotes a model trained on a dataset with SNR = *p* dB and evaluated on a test dataset with SNR = *q* dB. As observed in the figure, the detection performance remained nearly identical across models trained at different SNR levels when evaluated on the same test dataset. This indicates that our method successfully extracted signal-relevant information from the sample covariance matrix and was largely unaffected by variations in SNR. Consequently, ACM-CNN demonstrated strong generalizability and robustness to SNR mismatch between training and detection.

**Figure 16:**
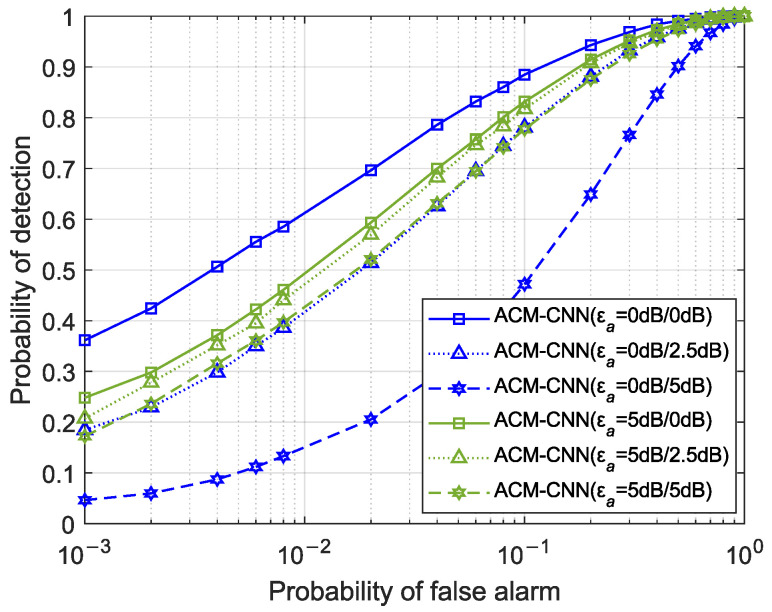
ROC curves with different degrees of antenna noise uncertainty between training and detection under N=160, SNR = −18 dB.

Figure 16 illustrates the ROC curves of the proposed ACM-CNN method with different degrees of antenna noise uncertainty between training and detection. Specifically, “ACM-CNN ($v_{a}\;\mathrm{dB}/\;w_{a}\mathrm{dB}$)” denotes a model trained on a dataset with $\varepsilon_{a}=v_{a}$ dB and evaluated on a test dataset with $\varepsilon_{a}=w_{a}$ dB. As observed in the figure, the neural network model trained under an antenna noise uncertainty of $\varepsilon_{a}=5$ dB performed slightly worse than the model trained with $\varepsilon_{a}=0$ dB when tested on a dataset with ideal uniform noise ($\varepsilon_{a}=0$ dB). However, when evaluated on test sets with nonzero antenna noise uncertainty ($\varepsilon_{a}\neq 0$ dB), the $\varepsilon_{a}=5$ dB-trained model outperformed its counterparts trained under ideal conditions. This phenomenon indicates that, while the presence of unstable noise across antennas during training introduces interference that slightly degrades peak detection performance, it simultaneously enhances the robustness to noise uncertainty. Consequently, the model trained with an antenna noise uncertainty of $\varepsilon_{a}\neq 0$ dB exhibited slower performance degradation under mismatched antenna noise conditions, demonstrating superior generalization capability to inter-antenna noise variations.

**Figure 17:**
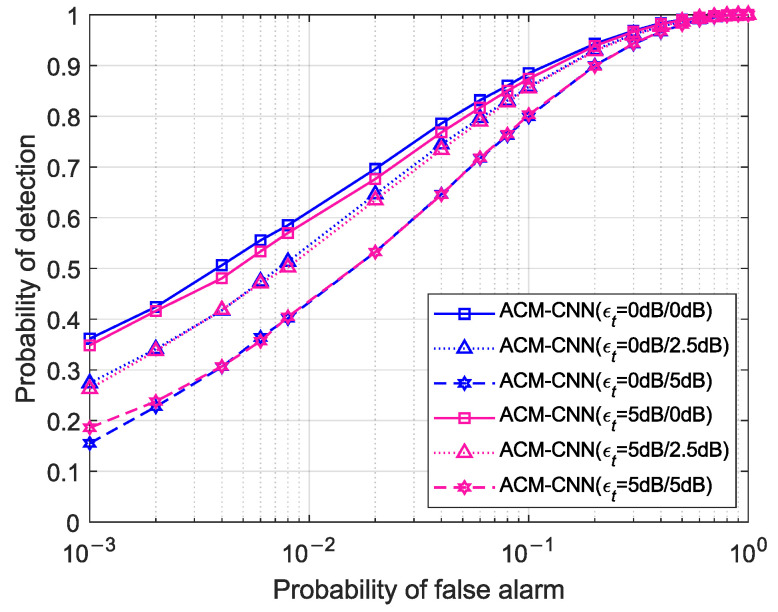
ROC curves with different types of noise uncertainty across time between training and detection under N=160, SNR =−18 dB.

Figure 17 illustrates the ROC curves of the proposed ACM-CNN method with different degrees of time noise uncertainty between training and detection. Specifically, “ACM-CNN ($v_{t}\;\mathrm{dB}/w_{t}\;\mathrm{dB}$)” denotes a model trained on a dataset with $\varepsilon_{t}=v_{t}$ dB and evaluated on a test dataset with $\varepsilon_{t}=w_{t}$ dB. Unlike the significant impact of unstable noise across antennas on model training, unstable noise across time had a much smaller effect on the training outcome and evaluation result. This is primarily because, prior to being fed into the neural network, the input data undergo covariance matrix computation, during which temporal variations are effectively averaged out. As a result, the influence of temporal noise uncertainty is substantially mitigated. These findings demonstrate that the proposed ACM-CNN method exhibits strong robustness and generalization capabilities in the presence of temporal unstable noise.

### 4.4. Probability of Detection Versus Noncircularity

**Figure 18:**
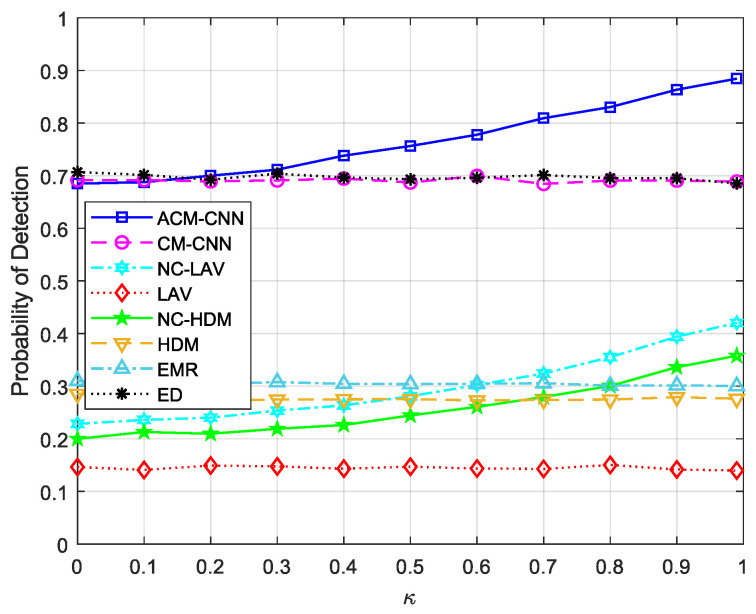
Detection probability versus noncircularity κ under $P_{f}=0.1$, N =160, $\varepsilon_{a}=0$ dB, $\varepsilon_{t}=0$ dB.

Similarly to existing non-circular signal-based methods, our proposed ACM-CNN leverages both the circular and noncircular components inherently present in such signals. In the special case of circular signals, where the noncircular component is zero, noncircular-signal-based methods naturally degenerate into their circular counterparts. To assess how different levels of noncircularity rate κ affect the performance of ACM-CNN, we present the detection probabilities of various algorithms as a function of κ, as illustrated in Figure 18. It can be observed that the performance of the three algorithms (ACM-CNN, NC-LAV, and NC-HDM) that exploit noncircular information improved significantly as κ increased, whereas those that do not utilize noncircular features remained unaffected by changes in κ. When the noncircularity rate κ approached zero, the performance of ACM-CNN closely aligned with that of CM-CNN. This demonstrates that ACM-CNN can gracefully reduce to a circular-signal-based model in the absence of noncircularity, while effectively leveraging additional structure when noncircularity is present, thus validating its adaptability and effectiveness for noncircular signals.

## 5. Conclusions

In this work, we have discussed the problem of spectrum sensing in multi-antenna cognitive radio networks by emphasizing the importance of non-circular signal characteristics. We proposed an augmented covariance-matrix-aware convolutional neural network (ACM-CNN) that integrates both conventional covariance and complementary covariance matrices into a unified learning framework. This design allows the model to fully exploit the second-order statistical features unique to non-circular signals, which are typically neglected in conventional spectrum sensing methods. Simulation results show that ACM-CNN significantly outperformed both traditional model-driven algorithms and CNN-based approaches in non-circular signal scenarios.

## Figures and Tables

**Table 1:** Hyperparameters of the augmented covariance-matrix-based CNN.

**Input:** Augmented covariance matrix array (16×16×2)	
**Layers**	**Size**
$C_{1}+$ ReLu	20@(3×3)
$C_{2}+$ ReLu	50@(3×3)
$F_{1}+$ ReLu	500×3200
$F_{2}+$ Softmax	2×500
**Output**: Score Vector (2×1)	

## Data Availability

Data are contained within the article.
